# Role of horizontally transferred copper resistance genes in *Staphylococcus aureus* and *Listeria monocytogenes*

**DOI:** 10.1099/mic.0.001162

**Published:** 2022-04-11

**Authors:** Inderpreet Kaur, Joanne Purves, Matthew Harwood, Julian M. Ketley, Peter W. Andrew, Kevin J. Waldron, Julie A. Morrissey

**Affiliations:** ^1^​ Department of Genetics and Genome Biology, University of Leicester, University Road, Leicester LE1 7RH, UK; ^2^​ School of Life Sciences, University of Warwick, Coventry, CV4 7AL, UK; ^3^​ Department of Respiratory Sciences, University of Leicester, University, Leicester, LE1 7RH, UK; ^4^​ Biosciences Institute, Newcastle University, Catherine Cookson Building Framlington Place Newcastle upon Tyne NE2 4HH, UK

**Keywords:** copper, *Staphylococcus aureus*, USA300, *L. monocytogenes*, CopA, CopX, CopL, plasmids

## Abstract

Bacteria have evolved mechanisms which enable them to control intracellular concentrations of metals. In the case of transition metals, such as copper, iron and zinc, bacteria must ensure enough is available as a cofactor for enzymes whilst at the same time preventing the accumulation of excess concentrations, which can be toxic. Interestingly, metal homeostasis and resistance systems have been found to play important roles in virulence. This review will discuss the copper homeostasis and resistance systems in *Staphylococcus aureus* and *Listeria monocytogenes* and the implications that acquisition of additional copper resistance genes may have in these pathogens.

## Introduction

Copper is an important transition metal in both prokaryotes and eukaryotes. Despite this, excess levels of copper can also be toxic and the delicate balance between ensuring enough copper is available as a micronutrient, whilst at the same time protecting from excess levels, plays a crucial role in the survival of many pathogens. *Staphylococcus aureus* has acquired various copper resistance mechanisms which have enabled this pathogen to survive in high concentrations of copper. Interestingly, several of the genes which confer copper resistance in *S. aureus* have also been identified in *Listeria monocytogenes* on plasmids, but the role of these homologous genes in *L. monocytogenes* has not been investigated. Evolutionary analyses to identify how these homologous copper resistance genes encoded on plasmids were acquired by *L. monocytogenes* suggest they have been horizontally acquired from staphylococci, which are known to share similar ecological niches with *L. monocytogenes *. In this review, the biological importance of copper and the role of mobile genetic elements in contributing to increased *S. aureus* copper resistance will be discussed, as well as the implications this may have in *L. monocytogenes *.

## The biological significance of copper

Copper plays a key role in many biological processes, as an essential micronutrient in both prokaryotic and eukaryotic organisms. A key feature of copper, as a transition metal, is its ability to cycle between reduced (Cu^+^) and oxidized (Cu^2+^) states, making it an excellent catalytic co-factor for several metalloenzymes (or cuproenzymes). Copper is essential for cellular metabolic processes such as respiration where the activity of cytochrome *c* oxidase, a copper-containing enzyme, plays a vital role as an electron acceptor in the last step of the electron transport chain.

### Copper toxicity

Despite its role as an important micronutrient in biological organisms, in excess levels copper is toxic. Although the precise molecular mechanisms of copper toxicity are not completely understood, several hypotheses have been proposed.

**Fig. 1.**
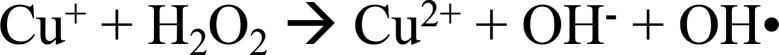
Copper-catalysed production of ROS may play a role in toxicity. Copper toxicity may occur through the production of hydroxyl radicals caused by excess free copper(I) ions oxidizing intracellular hydrogen peroxide. These hydroxyl radicals can cause cellular and DNA damage.

A long-standing hypothesis of how copper toxicity occurs is through oxidative damage. This has been attributed to the ability of copper to undergo redox cycling, such that in the presence of free intracellular hydrogen peroxide (H₂O₂), produced as a by-product of oxygen metabolism, Cu(I) catalyses the production of hydroxyl radicals (Fig. 1) [1]. The production of these reactive oxygen species (ROS) can be amplified in combination with the Haber–Weiss cycle and the presence of excess copper can act as a catalyst for these reactions. Hydroxyl radicals can cause DNA damage through the modification of DNA base breaks [2–4], as well as further cellular damage through attack of polyunsaturated fatty acids [3, 5]. This hypothesis has been supported by studies in *Escherichia coli *, where it has been found that copper stress causes induction of the *sox*RS regulatory system, involved in the superoxide stress response [6]. Furthermore, in *S. aureus *, copper stress has been shown to cause induction of the *S. aureus* protein misfolding and oxidative stress pathways [7].

Despite mounting evidence for this mode of copper toxicity, studies under anaerobic conditions have shown that copper is also toxic in the absence of oxygen [8, 9]. It has been suggested that the oxidative stress response caused by copper toxicity is not due to the direct participation of Cu^+^ in a Fenton-like reaction but may in fact be due to increased levels of Fe^2+^, one of many examples of intrinsic linkages between copper and iron metabolism in biology. In *E. coli,* copper ions can attack iron–sulphur clusters in dehydratases by copper liganding to cluster-coordinating sulphur atoms in an unknown manner, affecting cluster-dependent central biosynthetic and catabolic pathways [9]. Copper has also been shown to cause displacement of iron ions from iron–sulphur cluster assembly proteins, resulting in inhibition of iron–sulphur cluster biogenesis [10]. In the yeast *Saccharomyces cerevisiae*, damage to iron–sulphur clusters was identified as the main cause of copper toxicity due to several observations including loss of aconitase activity, increased activation of iron uptake and iron-regulons [11]. Furthermore, in *Bacillus subtilis,* copper stress was found to trigger upregulation of iron–sulphur cluster biogenesis as well as cause destabilization of the iron–sulphur cluster of SufU, a major scaffold protein required for iron–sulphur cluster assembly and transfer to target proteins [12]. As well as iron–sulphur clusters, copper toxicity can also occur through binding of copper to cytosolic proteins, such as the glyceraldehyde-3-phosphate dehydrogenase GapA, affecting their function [13].

### Copper as an antibacterial weapon within the animal host

**Fig. 2.**
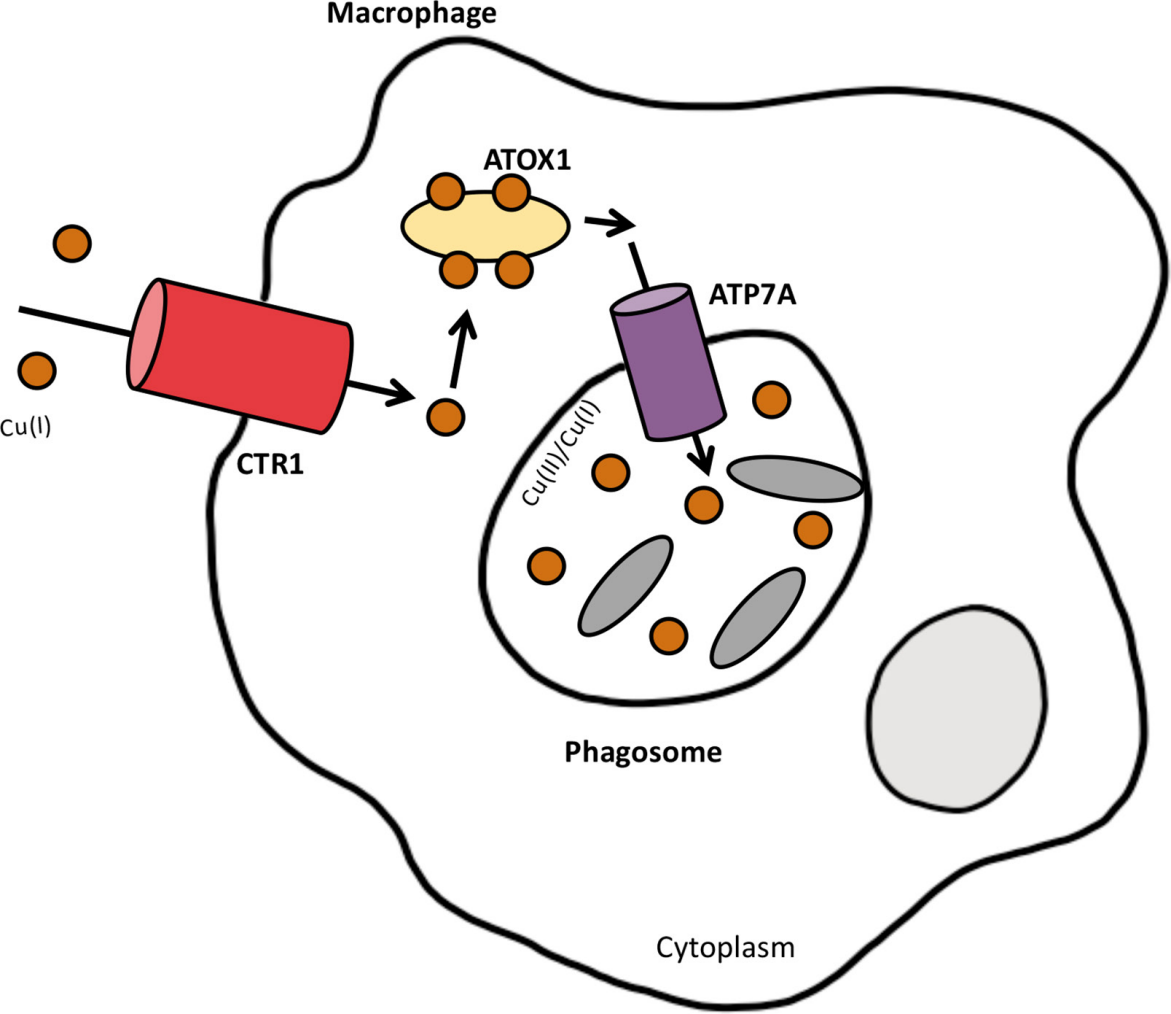
Copper is used as an antibacterial defence in macrophages. The high-affinity copper transporter, CTR1, transports copper across the plasma membrane of macrophages. Copper is then transported to ATP7A by the copper chaperone ATOX1. When macrophages are stimulated by IFN-γ, cytoplasmic vesicles that contain ATP7A fuse with phagosomes. Copper is transported to the phagosome, and this contributes to copper-dependent bactericidal innate immunity. Bacteria contained within the phagosome are denoted by the grey ovals and the nucleus is shown in light grey.

The animal host uses the toxic biochemistry of copper to its advantage as an important antibacterial defence, with copper being shown to accumulate at sites of inflammation [14]. The innate immune response to invasion by microorganisms involves humoral effectors, such as cytokines, and an arsenal of immune cells which include phagocytic cells such as macrophages. Macrophages can engulf microorganisms and contain them inside a highly hostile environment within a phagolysosome, formed by the fusion of a phagosome-containing microorganism with lysosomes. Phagosomes recruit the vacuolar ATPase, which is important for pumping of protons into the phagosomal lumen, causing acidification of the phagosome [15]. Phagosomes also recruit the NADPH oxidase complex, which catalyses the production of ROS in what is called a respiratory burst [15]. When fusing with the lysosomes to form a phagolysosome, hydrolytic enzymes such as DNAses, proteases and lipases contained within the lysosome are acquired. Together, this causes microbial killing. Copper has also been implicated in this process of microbial killing, with macrophages being found to have increased copper concentrations upon treatment with LPS and the proinflammatory agent IFN-γ, as well as infection with bacteria such as *Mycobacterium avium* and *Salmonella* Typhimurium [16, 17]. This increase in macrophage copper concentration occurs due to an increase in the expression of CTR1, a high-affinity copper transporter located at the macrophage plasma membrane, as well as upregulation of ATP7A, a copper transporter which is trafficked to the phagolysosomal compartment and is responsible for the uptake of copper into secretory vesicles (Fig. 2) [18]. The uptake of copper into the phagosome has been shown to be important for the bactericidal killing of *E. coli* [18], *Streptococcus pneumoniae* [19] and *Mycobacterium tuberculosis* [20]. It is believed that this copper-facilitated microbial killing occurs through the Fenton-type reaction described earlier, with copper ions reacting with hydrogen peroxide probably formed through the dismutation of superoxide radical anions generated from molecular oxygen by the NADPH oxidase integral membrane protein [21]. Other proposed models for the mechanism of copper-facilitated microbial killing include delayed vesicular accumulation of copper which can increase oxidative stress [17], as well as copper-induced FPN-1-dependent iron export which can starve intracellular bacteria of essential iron [22].

Evidence for increased copper concentrations at sites of infection has been supported by many studies. Higher concentrations of copper accumulate in the lungs of mice following infection with *Streptococcus pneumoniae* [23]. In guinea pigs infected with *Mycobacterium tuberculosis *, copper was found to accumulate in isolated primary granulomatous lung lesions [20]. It is likely that the elevated concentrations of copper found in the lungs can be attributed in part to the presence of macrophages, of which pulmonary macrophages are known to form a major part of tuberculosis granulomas [24]. In addition to macrophages, increased copper has also been observed in serum, as observed in mouse models of infections with *Candida albicans* and *Cryptococcus neoformans* [25, 26]. Furthermore, in pulmonary *Mycobacterium tuberculosis* patients an increase in the copper-containing multicopper oxidase, ceruloplasmin, has been observed in serum [27]. It has been proposed that ceruloplasmin may deliver copper to sites of infection for defence against pathogens, but due to its major role in iron metabolism, an example of the link between iron and copper in biology, it has also been suggested that ceruloplasmin depletes tissues of iron as a way to starve invading pathogens [28].

### Copper in the environment

Beyond the utilization of copper by the host to defend against invading microorganisms, bacteria can also encounter high concentrations of copper in the environment. Natural environments are known to be naturally contaminated with heavy metals caused by, for example, volcanic eruptions and forest fires. However, anthropogenic activity has led to further widespread environmental heavy metal contamination, largely due to urbanization which has caused the release of heavy metals such as copper into the air and industrialization which has caused the release of industrial effluents containing heavy metals into fresh water [29]. Soils are major sinks for heavy metal contamination, with rainwater causing heavy metals released into the air to be captured in the soil. This is further exacerbated by the application of heavy metal-containing animal manures, fertilizers and pesticides [29–31]. The fungicide known as Bordeaux mixture, one of the main ingredients of which is copper sulphate, is heavily applied in vineyards and organic agricultural settings, and the use of Bordeaux mixture on a mango orchard has been correlated with an increase in copper-resistant bacteria [32]. Due to the antimicrobial effects of copper and evidence that copper promotes growth rate and reduces the incidence of mortality in weanling pigs, it has often been used as an animal feed supplement in livestock at dietary (copper requirement of piglets is 4–6 mg Cu kg feed^–1^ [33]) and pharmacological doses (150–250 mg Cu kg feed^–1^) as an alternative to antibiotics [34–37]. The use of pharmacological doses of copper means the remaining unabsorbed copper is released, and the resulting pig slurry used as manure contains increased concentrations of copper, consequently resulting in accumulation of copper in agricultural soils.

Worryingly, studies have shown that the supplementation of pig feed and cattle feed with copper at higher than the physiological requirements are correlated with an increased prevalence of copper-resistant faecal enterococci, which also show a co-selection for macrolide resistance [38, 39]. This co-resistance between metals and antibiotics has also been identified in agricultural soils [40]. In *L. monocytogenes *, a penicillin binding protein (Pbp4) important for antibiotic resistance has been linked to copper tolerance [41].

## *Staphylococcus aureus* and copper homeostasis

The presence of copper in the environment and within the host poses a large risk to bacterial survival. Both Gram-negative and Gram-positive bacteria have evolved mechanisms which enable them to maintain copper homeostasis and survive within copper-containing environments. For the purposes of this review, the copper homeostasis mechanisms of the Gram-positive bacterium *S. aureus* will be discussed.

### S. aureus

*S. aureus* is a Gram-positive bacterium, known to be an asymptomatic commensal in healthy individuals residing permanently in the nasopharynx of approximately 30 % of the population and is also a component of the normal skin microflora [42]. Despite this, *S. aureus* is an opportunistic pathogen responsible for causing diseases ranging from superficial infections, such as acne and boils, to more invasive and life-threatening infections, such as osteomyelitis and endocarditis [43, 44]. Some *S. aureus* strains have acquired the staphylococcal cassette chromosome *mec* (SCC*mec*), which encodes the methicillin resistance gene, *mecA* [45], and has led to the emergence of methicillin-resistant *S. aureus* (MRSA). MRSA is particularly prevalent within nosocomial settings [described as healthcare-associated (HA)-MRSA)] due to its ability to infect surgical sites and form biofilms on surfaces and indwelling prosthetic devices [46].

### *S. aureus* core copper resistance machinery

**Fig. 3.**
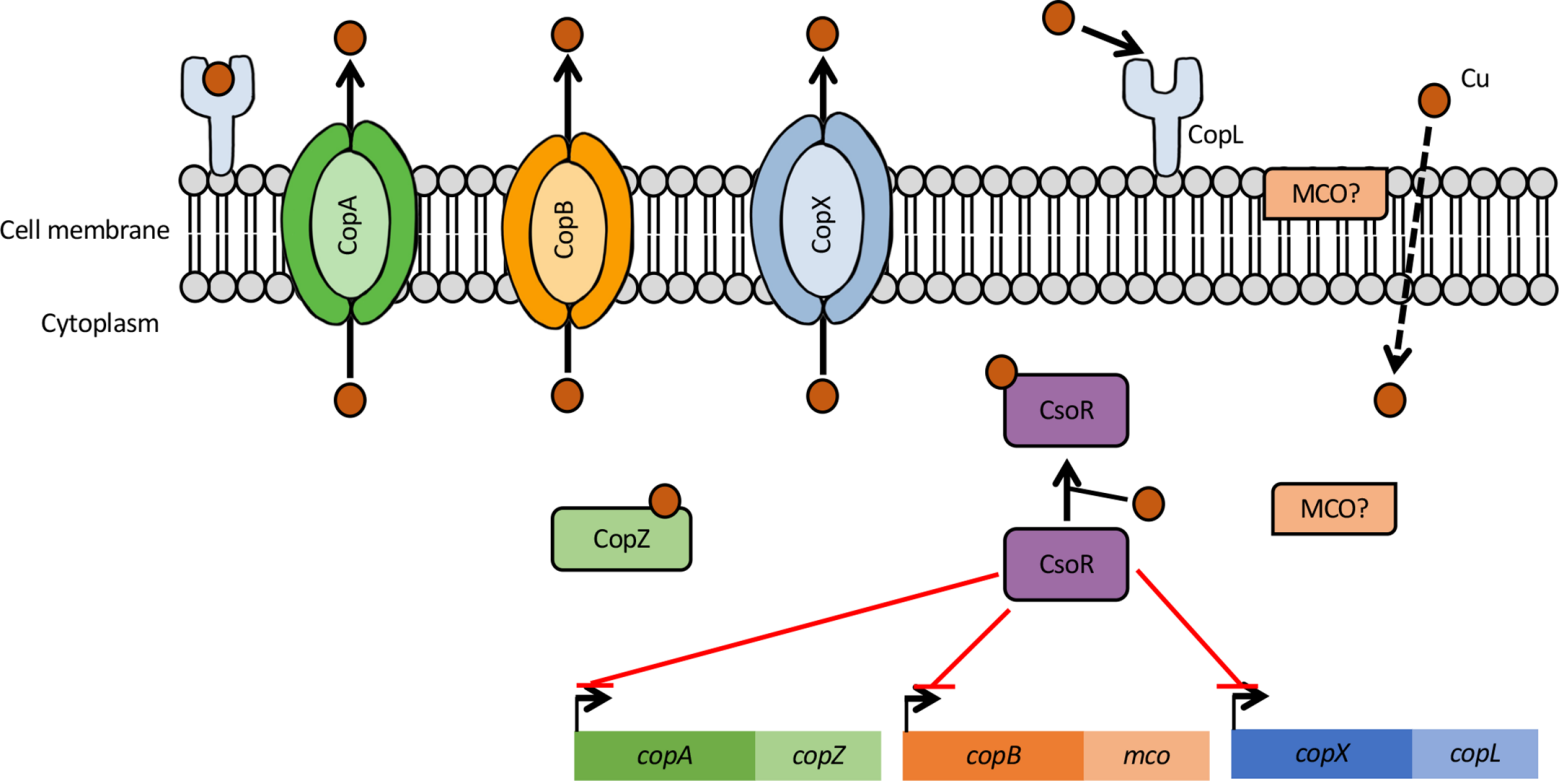
Summary of *S. aureus* copper resistance mechanisms. The *copAZ* operon encodes the core conserved copper resistance machinery of all *S. aureus* and consists of a P_1B-1_-type ATPase copper efflux transporter (CopA) and a small copper chaperone protein (CopZ). Some *S. aureus* strains have been found to carry the *copBmco* locus, either encoded on a freely replicating plasmid or integrated in the chromosome. *copBmo* encodes a P_1B_-type ATPase copper efflux transporter (CopB) and a multicopper oxidase (MCO). MCO may be located within the cytoplasm or associated with the cytoplasmic membrane/extracellularly. Some *S. aureus* strains have also been identified to carry the *copXL* locus, encoding a P_1B-3_-type ATPase copper transporter (CopX, but also described as CopB by others [53]) and a lipoprotein (CopL) of unknown function. It has been suggested that CopL acts to prevent copper uptake by binding extracellular copper. These operons are under the control of the copper sensitive operon repressor, CsoR, which is encoded by the *csoR* gene located separately from the copper resistance operons and in the absence of copper represses *copAZ*, *copBmco* and *copXL* through promoter binding [7, 47, 52–54].

**Fig. 4.**
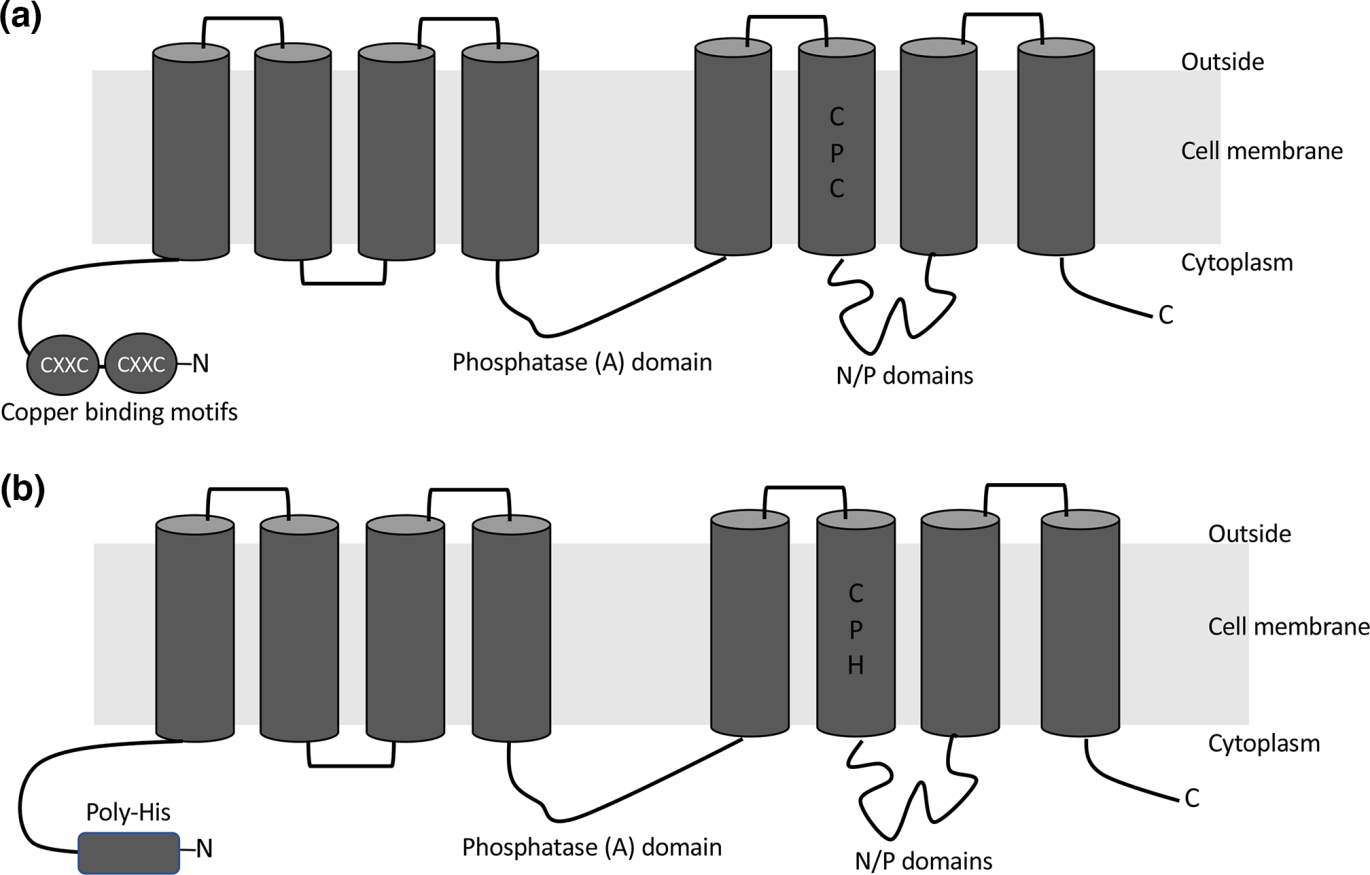
Schematic of *S. aureus* CopA, CopX and CopB P_1B_-type ATPase transporters. (a) The *copA* gene encodes a P_1B-1_-type ATPase copper efflux transporter (CopA) composed of eight conserved transmembrane domains and two amino terminal domains, each of which possess CxxC copper binding motifs [52, 97]. (b) The *copB* and *copX* genes encode a P_1B-3_-type ATPase (CopB and CopX, respectively) which is composed of eight transmembrane domains and an amino-terminal poly-His sequence. Alignment of these two transporters suggest they are different as they share 83 % overall sequence identity overall but only 59 % identity when only the His-rich N-terminal domains are aligned [52, 53]. Fig. 2A adapted from [52] published under a CC-BY license.

All *S. aureus* strains carry a core, conserved copper tolerance locus, *copAZ,* on their genome which encodes a P_1B-1_-type ATPase copper efflux transporter (CopA) and a small, 7.2 kDa copper chaperone protein (CopZ) (Fig. 3) [47]. CopA is composed of eight transmembrane domains and two cytoplasmic heavy metal binding domains that contain copper-coordinating (CXXC) motifs (Fig. 4a) and has been shown to efflux copper ions from the cytoplasm [47]. *S. aureus copA* mutants exhibit increased sensitivity to copper, iron and lead, as well as hydrogen peroxide which may be produced as a result of increased accumulation of copper within the cell [47]. The CopZ copper chaperone probably scavenges intracellular copper, delivering it to partner proteins and may also interact with CopA, as shown in *Enterococcus hirae* [47, 48].

### Regulation of copper homeostasis genes

The *S. aureus copAZ* operon is regulated by the copper sensitive operon repressor, CsoR, which in the absence of copper represses *copAZ* through binding to a putative G/C pseudo-inverted repeat region found in the promoter region (ATACCtataGGGGGTAC) [7, 49]. This repression in low copper conditions is lifted upon binding of Cu^+^ to CsoR with high affinity [7, 49]. It has been proposed that repression of *copAZ* is achieved through the binding of CsoR to a conserved inverted repeat DNA sequence located in the *copAZ* operator region, and Cu^+^ binding to CsoR is thought to induce conformational changes in the repressor that reduce its affinity for DNA binding [50, 51].

### Additional copper resistance machinery in *S. aureus* encoded on mobile genetic elements

In addition to the conserved core copper homeostasis genes, *copAZ*, found in all *S. aureus,* some strains of *S. aureus* have also been identified to carry additional copper tolerance genes which confer higher levels of copper resistance compared to strains which do not carry these genes [7, 52, 53].

### copBmco

The P_1B-3_-type ATPase copper efflux transporter (CopB) and a multicopper oxidase (MCO) encoded by the *copBmco* operon (Fig. 3) has been found in 34.4 % of all publicly available *S. aureus* genomes [54]. Similar to CopA, CopB also has the characteristic eight transmembrane domains, but instead of the two heavy metal binding domains with CXXC motifs found in CopA, CopB possesses a predicted intracellular amino-terminal histidine-rich metal binding domain, characteristic of P_1B-3_-type transporters (Fig. 4) [52, 55].

This locus has been found to be encoded either on a plasmid integrated into the chromosome or on a freely replicating plasmid [7, 56]. Like the *copAZ* operon of *S. aureus, copBmco* is also regulated by the CsoR transcriptional repressor in a copper-dependent manner with CsoR binding to a putative reverse complement consensus sequence (CCataatGGGGATAGG) [7]. Interestingly, complete repression of *copBmco* by CsoR has only been observed in chromosomally encoded *copBmco*; CsoR does not fully repress the plasmid-encoded *copB*. It has been proposed that this may be due to an inability of CsoR to diffuse to the plasmid-encoded *copBmco* promoter or, alternatively, that not enough copies of the repressor protein are available for complete repression when multiple operator sites are present in the cell [7, 54].

This operon has been implicated in copper resistance, conferring survival in toxic concentrations of copper as well as playing a role in the oxidative stress response [7, 57]. Furthermore, upregulation of both *copB* and *mco* were observed in *S. aureus* strains following infection of macrophages, suggesting that these genes may play an important role in the persistence of *S. aureus* within the host [54]. This locus was identified in 17.9 % of clinical invasive MRSA isolates from European hospitals [54], although whether these isolates carrying *copBmco* genes are associated with poorer health outcomes remains to investigated. The *copBmco* locus has also been found to be widespread amongst the livestock-associated (LA)-MRSA CC398, which is the most common clone found in European livestock, suggesting that the incorporation of copper into livestock feed may select for copper resistance by MRSA [54].

### *copXL* in CA-MRSA USA300

Human community-acquired MRSA (CA-MRSA) are most typically associated with skin and soft tissue infections (STTIs), respiratory infections and trauma wounds [58]. These MRSA appear to have taken a step further in their virulence capabilities, being able to spread within the community and infect healthy individuals who had no prior exposure to healthcare situations [59].

USA300 is an epidemic CA-MRSA strain which arose in the early 2000s, with initial cases isolated to risk groups such as individuals in prisons, as well as athletes [60, 61]. However, this strain began to spread rapidly across the USA [62], overtaking the USA400 CA-MRSA strain, becoming a major cause of SSTIs in the USA and also spreading globally to Europe [63, 64]. Following the identification of the epidemic USA300 clone in the USA, in 2005 similar incidences of infection were also emerging in South America, leading to the identification of the USA300 South American variant (USA300-SAE), which shares a recent common ancestor with the North American USA300 but lacks a mobile genetic element (ACME) [65]. The success of the North American USA300 (USA300-NAE) globally and USA300-SAE in South America has been attributed to the acquisition of the arginine catabolic mobile element (ACME) present in USA300-NAE and the copper and mercury resistance (COMER) mobile elements found in USA300-SAE [65, 66] respectively. Although the USA300-NAE and USA300-SAE variants were identified to have different mobile genetic elements, the epidemic capabilities of these strains were very similar and, interestingly, comparison of the ACME and COMER elements found in the USA300-NAE and USA300-SAE, respectively, led to the identification of just two conserved genes, *copXL*.

**Fig. 5.**
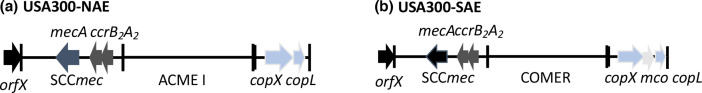
The ACME and COMER elements of USA300-NAE and USA300-SAE. The location of the conserved *copX* and *copL* genes (blue) identified in USA300-NAE and USA300-NAE. The USA300-NAE (a) differs from USA300-SAE (b) due to the presence of a different mobile genetic element, ACME. Comparison of these epidemic strains has shown they both carry the *copX* and *copL* genes encoded in the ACME and COMER elements. Fig. 1C taken from [52] published under a CC-BY license.

This *copXL* locus encodes a P_1B-3_-type ATPase copper efflux transporter (CopX) and a lipoprotein (CopL) of unknown function, and in the USA300 strain JE2 was found to be under the control of the CsoR transcriptional repressor with a putative CsoR binding site identified upstream from CopX (ATACCctggGTGGGTAT) (Fig. 3) [52, 53]. The P_1B-3_-type ATPase copper efflux transporter has been described as CopB or CopX by different authors [52, 53] as this transporter has been found to also possess a predicted intracellular amino-terminal histidine-rich metal binding domain, like CopB (Fig. 4b) [52]. However, it shares only 83 % sequence identity with CopB, and only 59 % identity when just the His-rich N-terminal domains are aligned [52, 53]. Although the function of CopL is unknown, it has been suggested that CopL may act to bind extracellular copper to prevent uptake or reuptake into the cell [53]. In South American isolates, a multicopper oxidase is also found encoded between the *copX* and *copL* genes (Fig. 5). It has been suggested that the *copXL* locus was recently acquired by USA300 from coagulase-negative *Staphylococcus* species by horizontal gene transfer [52]. Although these genes are not found in typical methicillin sensitive *S. aureus* (MSSA), *copXL genes* which show 99 % DNA sequence identity to the USA300 *copXL* have been identified in some clinical isolates, such as the human- and livestock-associated CC398 [52].

Interestingly, the presence of a single copy of *copXL* in USA300 has been shown to confer the same levels of copper hyper-resistance that were observed in *S. aureus* ATCC 12600, which encodes CopB on a multi-copy plasmid [52]. The importance of this *copXL* locus was further highlighted when it was found that both CopX and CopL play an important role in intracellular survival of *S. aureus* within macrophages, whereas CopA does not [52], suggesting that the recent evolution and success of USA300 may be due to possession of these additional copper resistance genes, enhancing bacterial fitness through increased resistance to copper-dependent bactericidal innate immunity.

## Copper homeostasis and resistance genes in *L. monocytogenes*

Similar to *S. aureus,* all strains of *L. monocytogenes* carry a highly conserved *csoR-copA-copZ* copper resistance operon. Interestingly, some strains of *L. monocytogenes* have been identified to carry additional predicted copper resistance genes on plasmids, which show high identity to *Staphylococcus* copper resistance genes. These plasmids in *L. monocytogenes* have been found to harbour multiple metal resistance genes predicted to also encode resistance to cadmium, zinc and arsenic [67–70]. Furthermore, many of the strains harbouring these plasmids have been associated with food, food processing and clinical outbreaks [71, 72]. In this section, the core copper homeostasis genes of *L. monocytogenes* will be discussed along with the identification of additional plasmid-encoded copper resistance genes which show high identity to *S. aureus copBmco* and *copL* and the role these may have in *L. monocytogenes* survival.

### Listeria monocytogenes

*L. monocytogenes* is a Gram-positive, catalase-positive, rod-shaped bacterium that is ubiquitous in the environment [73]. It is also an intracellular food-borne pathogen that can infect mainly immunocompromised individuals, newborn babies and pregnant women causing listeriosis [74]. This bacterium was first isolated in 1926 following the investigation of an epidemic infection of the livers of rabbits and guinea-pigs in an animal breeding unit and the first human case was also reported in the same year [75, 76]. In the 1980s, *L. monocytogenes* was identified as a major food-borne pathogen causing several outbreaks of listeriosis in humans [77]. Although the incidence of *L. monocytogenes* infection is not high, there is a high mortality rate even with adequate antibiotic treatment, with 30.3 % of non-pregnancy-associated listeriosis cases resulting in death in the UK in 2017[78].

### Core copper resistance machinery of *L. monocytogenes*

All *L. monocytogenes* genomes carry a core, conserved chromosomal copper resistance operon, *csoR-copA-copZ*. This *csoR-copA-copZ* operon encodes a P_1B-1_-type ATPase copper efflux transporter (CopA) and a copper metallochaperone (CopZ), and like *S. aureus,* is regulated by the CsoR DNA-binding copper-responsive repressor [79, 80]. Alignments of the *S. aureus* and *L. monocytogenes* CsoR share 36.5 % overall identity. Interestingly, unlike *S. aureus *, the CsoR transcriptional repressor does not completely dissociate from the *csoR-copA-copZ* operon of *L. monocytogenes* but upon copper binding changes conformation to allow for transcription of the operon [80]. *L. monocytogenes copA* mutants have been shown to exhibit increased copper sensitivity and increased copper accumulation, consistent with an analogous function to *S. aureus* CopA in copper efflux and demonstrating the importance of this operon for *L. monocytogenes* survival in the presence of copper [80].

### Identification of *copBmco* and *copL* in *L. monocytogenes* plasmids

Various plasmids in *L. monocytogenes* have been found to encode a predicted coppertranslocating P-type ATPase (LmCopB) (WP_019169200.1) and a multicopper oxidase (LmMco) directly downstream [67, 70] (Table 1). An alignment of the *S. aureus* ATCC 12600 CopB and Mco with the predicted plasmid-encoded LmCopB and LmMco showed 99.9 and 76.1% sequence identity, respectively. Furthermore, on some plasmids of *L. monocytogenes,* CopL (LmCopL) (WP_012952127.1) has been identified (Table 1), in addition to LmCopB and LmMco [70], that shows 66.7 % identity to the USA300 JE2 CopL [53] and >99 % identity to the CC398 LA-MRSA CopL [52, 53, 70]. Such high levels of sequence homology of the *S. aureus* proteins with the *L. monocytogenes* plasmid-encoded proteins may even imply direct transfer of these genes between these, or at least very closely related organisms.

**Table 1.** List of 18 plasmids from the Refeq database composed of 52 fully sequenced *L. monocytogenes* plasmids identified to carry predicted copper resistance genes. Shaded areas indicate presence within a genome.

	Gene			
Strain	*copB*	*mco*	*copL*	Accession number
AT3E				NZ_CP015509.1
MF4626				NZ_CP025083.1
HPB5415				NZ_CP019166.1
Lm N1546				NZ_CP013725.1
LMP18-H8393				NZ_CP041214.1
N1-011A				NC_022045.1
PIR00545				NZ_CP025561.1
SLCC2372				NC_018889.1
R479a				NZ_HG813248.1
SLCC2482				NC_018888.1
SLCC2755				NC_014495.1
R2-502				NC_021828.1
PIR00540				NZ_CP025569.1
2015TE24968				NZ_CP015985.1
HPB5622				NZ_CP019168.1
08–5578				NC_013767.1
198				NZ_CM008329.1
AUSMDU00000224				NZ_CP045973.1

blast searches of these predicted copper tolerance genes have suggested that they are widespread amongst *L. monocytogenes* plasmids. However, from an extensive search of the literature it appears that the roles of these plasmid-encoded copper resistance genes have not yet been explored in *L. monocytogenes *. From conducting evolutionary analyses to identify how these genes on plasmids in *L. monocytogenes* were acquired and from where, we found these genes were most likely horizontally acquired from staphylococci, which are known to share similar ecological niches with *L. monocytogenes* including soils and water [81–83].

### Implications for the acquisition of *Staphylococcus* copper resistance genes in *L. monocytogenes* survival in the environment, in food processing facilities and in the host

The ability of *L. monocytogenes* to acquire a vast array of genes as part of its accessory genome contributes to its ability to adapt to harsh natural and food processing environments. Specifically, plasmids appear to play a major role in the survival of *L. monocytogenes* in food processing environments, conferring resistance to acid stress, salt stress and temperature [84–86]. Furthermore, plasmids have been found to be overrepresented amongst *L. monocytogenes* isolated from foods. Therefore, the distribution of loci highly similar to *Staphylococcus* copper resistance genes amongst plasmids of *L. monocytogenes* is of particular interest. This combined with the changing epidemiology of *L. monocytogenes *, with more outbreaks being observed in novel food vehicles, thought to be associated with the entry of *L. monocytogenes* into food processing facilities from the environment [87–89], suggests that these plasmids carrying resistances to heavy metals may play an important role in the success of *L. monocytogenes *.

Price and colleagues found that although the key virulence factors of *L. monocytogenes, prfA* and *hly,* play an important role in biofilm formation and aggregation in strain 2011 L-2858 implicated in the 2011 cantaloupe outbreak, these virulence factors did not appear to be key virulence determinants for the colonization of fresh produce [87, 90]. This suggests that other factors play an important role in explaining the increase in outbreaks and the ability of *L. monocytogenes* to colonize novel food vehicles. Soils are a known ecological niche of *L. monocytogenes,* and therefore the acquisition of these predicted additional plasmid-encoded copper resistance genes could provide a selective advantage for survival of *L. monocytogenes* in the environment. Copper is also used an as antibacterial in animal feed, so these metal resistance genes may provide a secondary advantage and enable these isolates to colonize and infect farm animals, similar to *S. aureus *, which in turn results in the release of *L. monocytogenes* back into the soil and crops through faeces and the use of animal manure for fertilization.

The introduction and persistence of *L. monocytogenes* into food processing facilities is probably due to introduction of contaminated raw materials and/or ineffective sanitation or movement of people/equipment [91]. It has been suggested that these plasmids carrying heavy metal resistance genes may confer a selective advantage to the survival of these bacteria within food processing facilities through resistance to disinfectants [67]. Mullapudi and colleagues found that plasmid-encoded cadmium-resistant *L. monocytogenes* isolated from turkey processing plants exhibited resistance to quaternary ammonium disinfectants. This suggests that, in addition to conferring cadmium resistance, which would enable survival in cadmium-contaminated environments, cadmium resistance genes may either also confer resistance to disinfectants or be co-selected with genes associated with resistance to disinfectants [92, 93].

As a facultative intracellular pathogen, the acquisition of *copB, mco* and *copL* on plasmids by *L. monocytogenes* from *Staphylococcus* may also confer a selective advantage in these strains through increased survival against antibacterial copper produced by the host innate immune system [94, 95], but there appears to be no known literature on the role of these genes in *L. monocytogenes *. In *L. monocytogenes* CopA was not shown to be important for the virulence of *L. monocytogenes* in mice [80]. Furthermore, the intracellular lifestyle of *L. monocytogenes* is quite distinct from *S. aureus* in that this pathogen is capable of rapidly escaping from macrophage phagosomes [96]. Therefore, it should be noted that these additional predicted copper resistance genes in *L. monocytogenes* may provide little to no advantage in survival against macrophage copper as early phagosomal escape would avoid the need for copper resistance mechanisms in macrophages.

**Fig. 6.**
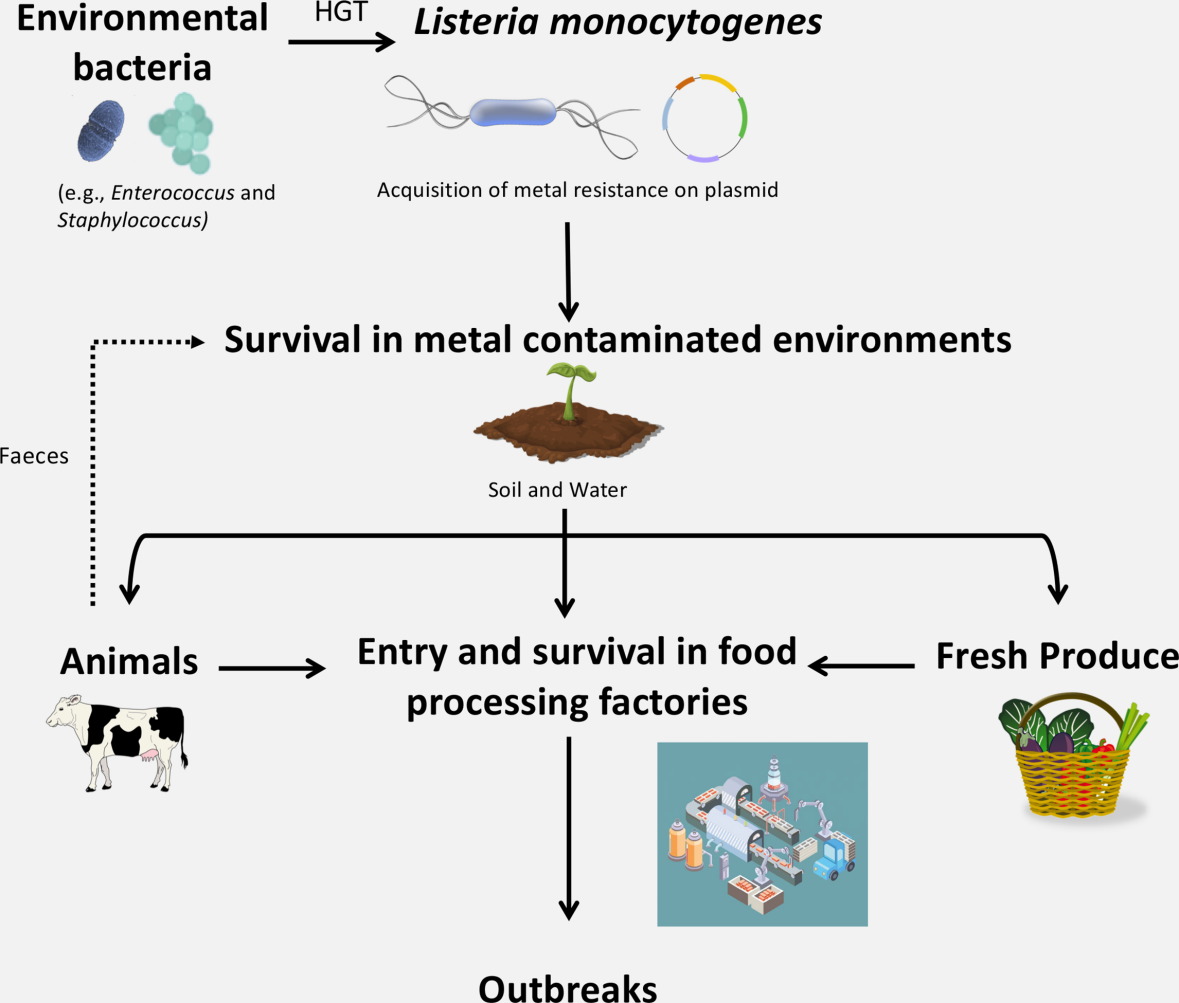
Acquisition of metal resistance genes by *L. monocytogenes* may confer advantages in the environment and within food processing facilities. *L. monocytogenes* has acquired metal resistance genes on plasmids. These metal resistance genes may confer a selective advantage to *L. monocytogenes* in metal-contaminated environments, such as soil and in metal-containing animal feed (where copper is known to be added). The introduction of *L. monocytogenes* into food processing facilities is probably due to introduction of contaminated raw materials and/or ineffective sanitation or movement of people/equipment [91].

To summarize, we hypothesize that the acquisition of additional plasmid-encoded copper resistance genes from *Staphylococcus* to *L. monocytogenes* probably initially conferred a selective advantage within the environment, which is known to be contaminated with high concentrations of heavy metals (Fig. 6). It is possible that these genes also confer secondary selective advantages within food processing facilities and confer increased virulence within hosts, potentially explaining the increase in *L. monocytogenes* outbreaks observed in recent years (Fig. 6). However, further research would be required confirm this.

This review aimed to discuss the copper homeostasis and resistance systems in *S. aureus* and *L. monocytogenes *. The acquisition of the additional copper resistance genes, *copB, mco, copX* and *copL* in *S. aureus* have been shown to confer a selective advantage to survival of *S. aureus* within macrophages. Furthermore, it is possible these genes also contribute to the survival of *Staphylococcus* within the natural environment, such as soils and water, of which copper is a major contaminant. Interestingly, many plasmids of *L. monocytogenes* have been identified to carry genes of high identity to copper resistance genes also identified in *S. aureus,* including *copB, mco* and *copL*. With the intracellular lifestyle of *L. monocytogenes* highly distinct from that of *S. aureus *, it is possible that these additional plasmid-encoded predicted copper resistance genes may not confer a selective advantage for the survival of *L. monocytogenes* within macrophages. However, the common environmental reservoirs shared between these pathogens may contribute to the exchange of such genes between species and also enable *L. monocytogenes* to survive within metal-contaminated environments. Further investigation into how these plasmid-encoded predicted copper resistance genes would benefit *L. monocytogenes* would provide clarity on the role of these genes in this important food-borne pathogen.
